# Survival Analysis Using Neoadjuvant Rectal Score in Locally Advanced Rectal Cancer Patients Who Underwent Surgery After Neoadjuvant Chemoradiotherapy: An Ambispective Study

**DOI:** 10.7759/cureus.86859

**Published:** 2025-06-27

**Authors:** Madhu Muralee, Akhil T Jacob, Chandramohan K, Paul Augustine, Arun Peter, Kurian Cherian, Mira Sudham, Sivanandan C D

**Affiliations:** 1 Surgical Oncology, Regional Cancer Centre, Thiruvananthapuram, Thiruvananthapuram, IND; 2 Surgical Oncology, Muthoot Cancer Centre, Kozhencherry, IND; 3 Surgical Oncology, Sree Mookambika Institute of Medical Sciences, Kanyakumari, IND; 4 Surgical Oncology, Lisie Hospital, Ernakulam, IND; 5 Radiation Oncology, Regional Cancer Centre, Thiruvananthapuram, Thiruvananthapuram, IND

**Keywords:** locally advanced rectal cancer, neoadjuvant chemoradiotherapy, neoadjuvant rectal score, overall survival, pathological complete response (pcr), surrogate predictor, trial end point

## Abstract

Purpose

The purpose of this study is to stratify patients into various risk groups based on their neoadjuvant rectal (NAR) score and study the predictive value of the NAR score in terms of oncological outcomes. This, in turn, can be used as a surrogate marker to prognosticate the patients and also to study the efficacy of various neoadjuvant therapeutic options.

Methodology

This ambispective observational study of patients who underwent long-course neoadjuvant chemoradiotherapy (NACTRT), followed by curative surgery in the form of anterior resection (AR) or abdominoperineal resection (APR), from 2010 to 2015 at a high-volume tertiary cancer center in South Kerala, with the survival data collected prospectively. The clinicopathological details of the patients were obtained. ypTNM status was recorded, and the NAR score was calculated. The patients were prospectively followed up for recurrence or death. All endpoints were calculated from the date of the start of NACTRT.

Results

Four hundred fifty-nine patients were included in the study, with 190 (41.4%) being women and 202 (44%) being less than 60 years old. Two hundred fourteen (46.62%) had lesions of 5-10 cm from the anal verge. Three hundred fifty-nine (78.21%) patients had T3 disease pre-NACTRT. Two hundred twenty-eight patients (51.85%) had undergone APR, and 369 (80.4%) had open surgery. Fifty-one patients (11.1%) had attained pathological complete response (PCR). One hundred twenty-eight patients (27.8%) had disease recurrence, of which 71 had distant metastasis. NAR was a significant predictor of overall survival (OS), disease-free survival (DFS), distant metastasis-free interval (DMFI), and locoregional relapse-free interval (LRFI). On the multivariate analysis of factors predicting survival, only NAR was found to be significant.

Conclusion

The NAR score is a significant predictor of OS, DFS, LRFI, and DMFI and can be used as a short-term surrogate for survival data in anticipated trials.

## Introduction

According to the global cancer statistics, as recorded by GLOBOCAN, colorectal cancer ranks third among the most common malignancies and is a major cause of cancer deaths [1]. Adopting the results from the landmark German CAO/ARO/AIO-94 trial, current guidelines for locally advanced rectal cancers (LARCs) recommend neoadjuvant chemoradiotherapy (NACTRT) as one of the preferred modalities to obtain better local tumor control and sphincter preservation [2]. Other alternatives are short-course radiotherapy (SCRT) and total neoadjuvant therapy (TNT), followed by surgery in the form of total or partial mesorectal excision (TME/PME). The optimal strategy for treating rectal adenocarcinoma is dependent on a number of deciding factors, of which the distance from anal verge and the local disease extent are of paramount importance.

To determine the impact of these management options on patients, at a trial level and in a real-world scenario, the usual endpoints are the survival outcomes. But these are not available at an early point in time during the evaluation, and hence, to determine if an intervention is efficacious or futile, there is a requirement for long-term evaluation.

Thus, there is a need for prognostic and surrogate markers, which would readily be available, in the short term, while being veritable in their measure of efficacy of an intervention, over the long-term follow-up. One such score was developed in the National Surgical Adjuvant Breast and Bowel Project (NSABP) R04 trial and validated in many subsequent studies, the neoadjuvant rectal score (NARS) [3]. NARS takes into account the pre-NACTRT T stage and N stage and the post-NACTRT pathological T stage and N stage to come up with the score.

The formula was designed from the National Surgical Adjuvant Breast and Bowel Project (NSABP) R-04 trial [3] and incorporated in the landmark CAO/ARO/AIO-04 trial [4]. It has been validated in clinical practice among the Asian population in a study by Lim et al. and published in the Diseases of the Colon & Rectum [5]. The neoadjuvant rectal (NAR) score uses variables routinely determined and readily available to investigators during the conduct of prospective studies or trials.

NARS is calculated, and the patients were then categorized into three groups, which are the low-risk category with a score of less than 8, the intermediate-risk category with a score of 8-16, and the high-risk category with a score of more than 16.

There is variability in the studies of the validity of NARS in various populations, which may be due to the deviation of the real-world population from the highly selected trial population or may even be due to ethnic variations and resultant change in the biology of the population, as well as the disease [5,6]. Thus, for this to be useful for the population of interest, it needs to be validated in the same population.

There are very limited data on the validity of NARS in the Indian population. This is the knowledge gap that this study aims to address. This study was done in a high-volume tertiary care oncological center in South India, with a protocol of NACTRT, followed by TME, which has been offered as the standard of care.

Aim

The aim of this study is to stratify patients into various risk groups based on their NAR score and study the predictive value of NARS in terms of oncological outcomes.

Primary objective

The primary objective of this study is to estimate the ability of NARS to predict overall survival (OS).

Secondary objectives

Other objectives of this study are to estimate the ability of NARS to predict disease-free survival (DFS); to assess the impact of clinicopathological factors such as distance from anal verge, clinical and pathological stage, mode of surgery, attainment of pathological complete response (PCR), positive lymph node ratio (PLNR), and reception of adjuvant therapy on survival outcomes; and to determine the recurrence pattern of carcinoma rectum.

This study was presented as a poster at the 43rd annual conference of the European Society of Surgical Oncology (ESSO) at Antwerp, Belgium, in October 2024. This has also been submitted as a preprint via Research Square.

## Materials and methods

This was done as an ambispective observational study in a tertiary care cancer center, among patients who underwent NACTRT, followed by TME, during the period of January 2010 to December 2015. The Scientific Review Committee, Institutional Review Board, Regional Cancer Centre, Thiruvananthapuram, issued approval 06/2021/07.

Inclusion criteria

The inclusion criteria of the study include biopsy-proven rectal adenocarcinoma (up to 15 cm from the anal verge), which was deemed to be locally advanced rectal cancer (LARC), which included T3 or T4 and/or node-positive malignancies. All patients were post long-course NACTRT. Further, all patients had curative intent rectal surgery with TME. The minimum follow-up period was three years.

Exclusion criteria

The following patients were excluded: patients with previous history of any other malignancies or second cancers during the follow-up period, patients with synchronous rectal colorectal cancers, patients with oligometastatic disease planned for curative surgery, patients lost to follow-up, patients who cannot be traced or contacted for follow-up, and patients whose records are incomplete and do not yield the required data.

All patients included in the study had undergone biopsy and staging with magnetic resonance imaging (MRI) of the pelvis and contrast-enhanced computed tomography (CECT) of the thorax and abdomen and carcinoembryonic antigen (CEA) prior to the initiation of neoadjuvant therapy. The patients underwent neoadjuvant chemoradiotherapy with capecitabine 1000-1250 mg per day and radiotherapy of 50.4 Gy in 25 fractions. All patients were evaluated with an MRI of the pelvis in the postoperative period. The surgery was done in the form of laparoscopic or open surgery and as abdominoperineal resection (APR) or anterior resection (AR), depending on the location of the tumor. Adjuvant therapy was given to patients who did not achieve a pathological complete response, with a regimen as per the International Duration Evaluation of Adjuvant Chemotherapy (IDEA) collaboration data. Patients were followed up with CEA every two months for the first two years, followed by every six months up to five years. Patients with elevated CEA above 5 ng/mL were evaluated with imaging in the form of CECT of the thorax, abdomen, and pelvis or positron emission tomography/computed tomography (PET/CT). Data on survival were recorded from January 2010 to December 2020 retrospectively and then followed up prospectively from January 2021 to August 2023.

Based on an article by Lim et al. with a level of significance fixed at 5% and power at 80%, the minimum sample size required for the present study was calculated to be 456 [5].

The demographic profile (age, sex, comorbidities, habits, and family history) of the patients was recorded. The clinicopathological data of the patients were collected and collated based on hospital records, images, and clinical follow-up. Suspected recurrences were biopsied if accessible. If the CEA was elevated and images were highly suspicious of malignancy, biopsy was not pursued.

Study endpoints

The study endpoints were as follows: overall survival (date of diagnosis to date of death), disease-free survival (date of diagnosis to date of recurrence), locoregional relapse-free interval (LRFI) (date of surgery to date of locoregional relapse), and distant metastasis-free interval (DMFI) (date of surgery to date of distant metastasis being diagnosed).

The Strengthening the Reporting of Observational Studies in Epidemiology (STROBE) guidelines for observational studies were adhered to.

Statistical analysis

The categorical variables were presented using frequency and percentages. The continuous variables were summarized using mean and standard deviation. The association between two categorical variables was assessed using the chi-square test. The survival probabilities were estimated using the Kaplan-Meier method, and the significance between the survival curves was tested using the log-rank test. The risk for survival was estimated using the Cox regression analysis. A p-value of less than 0.05 was considered to be significant. Multicollinearity was assessed using a scatter plot and corrected by Ridge regression.

## Results

Demographic data

Data from 459 patients were collected. It comprised 190 women and 269 men. The study population comprised 257 patients (56%) aged less than 60 years and 202 (44%) aged more than or equal to 60 years. The mean age of the sample population was 56.51 years, with the range from 24 to 82 years of age. Most of the patients had an Eastern Cooperative Oncology Group (ECOG) performance score of 0 or 1 (Table 1).

**Table 1:** Demographic and clinicopathological variables APR, abdominoperineal resection; AR, anterior resection; Lap, laparoscopic; CEA, carcinoembryonic antigen

Parameter	Subgroups	n	%
Sex	Male	269	58.60%
	Female	190	41.39%
Distance from the anal verge	Less than 3 cm	107	23.31%
	3-5 cm	97	21.13%
	5-10 cm	214	46.62%
	10-15 cm	41	8.94%
CEA value	<5 ng/mL	220	54.59%
	5-20 ng/mL	108	26.79%
	>20 ng/mL	75	18.61%
	Not recorded	56	-
Lap versus open	Lap	74	16.12%
	Open	369	80.39%
	Lap converted to open	16	3.49%
APR/AR	APR	238	51.85%
	AR	221	48.15%
cT	cT3	359	78.21%
	cT4a	49	10.68%
	cT4b	51	11.11%
cN	N0	43	9.37%
	N1	278	60.57%
	N2	138	30.07%
ypT	ypT0	56	12.20%
	ypT1	13	2.80%
	ypT2	155	33.80%
	ypT3	214	46.60%
	ypT4a	8	1.70%
	ypT4b	13	2.80%
ypN	ypN0	306	66.70%
	ypN1	117	25.50%
	ypN2	36	7.80%

Pre-NACTRT tumor and oncological status

With regard to the location of the tumor with relation to the anal verge, 214 patients (46.62%) had tumors 5-10 cm from the anal verge, 107 (23.31%) had tumors within 3 cm of the anal verge, and 97 (21.13%) had tumors 3-5 cm from the anal verge. Three hundred fifty-nine (78.21%) of the patients had T3 disease at presentation, followed by T4b status in 51 (11.11%). Two hundred seventy-eight (60.57%) of the population had N1 disease as per radiological assessment, and 138 (30.07%) had N2 disease. Most patients, that is, 220 patients (54.59%), had CEA of less than 5 ng/mL, prior to the commencement of NACTRT (Table 1).

Surgical aspects and postoperative histopathological data

In the study population, 238 patients (51.85%) had undergone APR, and 221 (48.15%) had undergone AR. With regard to the approach of surgery, 369 (80.39%) were done as open procedures, 74 (16.12%) were done laparoscopically, and 16 (3.49%) were started as laparoscopic procedures and converted to open. The proximal margins were free for all patients, but the distal margins were involved in four patients (0.9%). The circumferential resection margin (CRM) was involved in 25 patients (5.4%). Two hundred fourteen (46.62%) patients had ypT3 status; 155 (33.7%) had ypT2 status, while 56 (12.2%) had no residual lesion in the primary site. Three hundred six (66.67%) had ypN0 disease, 117 (25.49%) had ypN1 disease, and 36 (7.84%) had ypN2 disease. Fifty-one patients (11.1%) had attained PCR. The mean nodal yield was 7.13 nodes with a range from 1 to 24, with a skewed distribution. The mean positive lymph node ratio was 0.1192.

NAR score

The range of the NAR score was from 0 to 65, with the mean value being 15.97 and the mode being 8. Two hundred twenty-five (49%) of the patients belonged to the intermediate-risk category (category 2: score of 8-16), with 147 (32%) in the high-risk category (category 3: score of more than 16) and 87 (19%) in the low-risk category (category 1: score less than 8).

Follow-up and events

The mean follow-up period was 90.5 months, with the mode being 88 months. Four hundred forty-seven patients (97.4%) received adjuvant chemotherapy after surgery. Ten patients (4.52%) had developed anastomotic leaks, which were clinically apparent and required diversion or intervention in the form of drain insertion. One hundred twenty-nine patients developed disease recurrence, with the distribution being 37 (29%) locoregional recurrence alone, 71 (55%) distant metastatic recurrence alone, and 20 (16%) of both locoregional and distant recurrence. At the end of the follow-up period (August 2023), 313 (68.2%) were alive without disease, of whom two had metachronous metastasis and had undergone metastatectomy for the same. Five patients were alive with recurrent disease. One hundred seventeen patients had expired due to cancer (25.5%), while 24 (5.2%) had expired due to other causes (Table 2).

**Table 2:** Survival outcomes

Survival parameter	Mean	Standard error
Locoregional relapse-free interval (LRFI)	134.094	1.862
Distant metastasis-free interval (DMFI)	125.271	2.247
Disease-free survival (DFS)	115.932	2.527
Overall survival (OS)	118.987	2.130

NAR score versus overall survival (OS)

**Figure 1:**
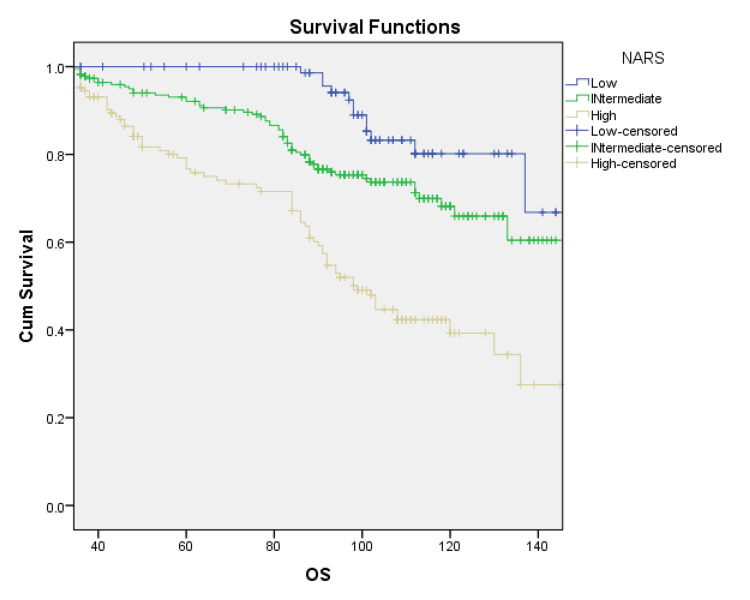
NARS versus overall survival: Kaplan-Meier curve NARS, neoadjuvant rectal score; OS, overall survival

The relation of neoadjuvant score with overall survival was analyzed. The categories of the NAR score were significantly found to be associated with overall survival when assessed with the Kaplan-Meier analysis (Table 3) (Figure 1). The events of death or loss to follow-up were used to censor the data.

**Table 3:** NARS versus seven-year OS: survival outcomes based on the category of NARS Survival analysis done by the Kaplan-Meier method and survival curves compared by the log-rank test method with the chi-square test NARS, neoadjuvant rectal score; OS, overall survival

Time (years)	Low-risk category		Intermediate-risk category		High-risk category			P-value
	Survival probability percentage	Standard error percentage	Survival probability percentage	Standard error percentage	Survival probability percentage	Standard error percentage	Chi-square	
7	87.3	3.6	78	2.8	55.8	4.1	46.051	<0.01

At the median follow-up of 84 months, the NAR low-risk category had a survival probability of 87.3%, which decreased to 78% in the intermediate-risk category and 55% in the high-risk category.

Factors impacting overall survival

The impact of factors such as comorbidities, pre-NACTRT CEA value, approach of surgery (laparoscopic versus open), circumferential resection margin (CRM) status, distal margin involvement status, pathological complete response (PCR) status, positive lymph node ratio (PLNR) (with 0.28 as cutoff), and history of clinically significant leaks were assessed, and positive CRM, PLNR, and PCR status was found to have statistically significant impact on overall survival. Patients in whom CRM was involved had 56% survival in comparison to 73.7% (at seven years) in patients without CRM involved (p=0.038). Similarly, PCR status was found to be positively correlated with survival (mean survival: 135 versus 116 months) (p=0.001). PLNR, with a cutoff value of 0.28, was significantly related to overall survival, with a mean survival of 123 months versus 94 months (p<0.0001). Pre-NACTRT T stage and ypN stage were also found to be statistically significant in their association with the OS, though they form components of the NAR score. The percentage of survival probability was calculated at seven years since >80% of the population had completed seven years of follow-up. On multivariate Cox regression analysis, only the NAR score had a significant value when PLNR, T status, pN status, and CRM were also included in the analysis.

Factors affecting DFS

Factors such as comorbidities, PCR status, pre-NATCRT CEA value, laparoscopic versus open procedure, CRM status, and distal margin status were analyzed for impact on disease-free survival, and PLNR, CRM status, and PCR status were found to have an impact on DFS. CRM-positive patients had a DFS probability of 52% at seven years versus 70.6% in the CRM-negative group. Gender was also found to be associated with DFS, with men having a longer DFS (p=0.035). Pre-NACTRT T and N stages and ypN stage were also found to have a statistically significant relation with the DFS. PCR was also found on univariate analysis to be significantly related to DFS (p=0.002). PLNR with a cutoff of >0.28 was also significantly associated with DFS, with the mean survival of 83 months versus 121 months in favor of those with lower PLNR (p<0.0001).

## Discussion

In the era of technologies evolving rapidly, the implementation of any treatment needs rigorous validation and rigorous evaluation using well-conducted trials. To derive a meaningful effect from treatment, the treatment modalities have to be beneficial in the long term. But the evaluation of these, taking 5-10 years to come up with a verdict of useful or futile, would put a great burden on the scientific community. This is especially true in a resource-limited society such as our own. In this scenario comes the relevance of a robust surrogate tool, which can accurately predict the long-term outcome using easily available variables. Here lies the role of the NAR score. In this study, we were able to prove that in the Indian population, the NAR score had a significant association with survival, including overall survival, disease-free survival, distant metastasis-free interval, and locoregional relapse-free interval. This indicates that the NAR score can be used as a viable surrogate in lieu of traditional oncological outcomes in the Indian population. On multivariate analysis, the NAR score maintained statistical significance over PLNR, PCR status, etc. Thus, rather than relying on individual factors, the NAR is still a more valid surrogate.

The gender distribution in the study was male predominant, similar to previously recorded Indian data [7]. Male patients were found to have a longer disease-free survival and distant metastasis-free survival, but there is no difference with regard to overall survival. This is not in keeping with the published data. This needs further studies to ascertain the cause, which may be biological or due to socio-economic and cultural variations.

There were more individuals less than 60 years old in the population studied. This contrasts with Western data, where there are more colorectal malignancies in the elderly population. Contrasting with the data from Tata Memorial Hospital, where just 22% of the population was more than 60 years, in our data, about 44% were older than 60 years of age [8]. This may be due to the slightly higher proportion of elderly population in the state of Kerala, compared to the whole of India [9,10].

Most of the tumors in the study population were about 5-10 cm from the anal verge. This was significantly related to the type of surgery, but even for low rectal cancers, post-NACTRT, sphincter preservation could be offered, albeit in lower numbers.

Pre-NACTRT CEA value was evaluated for its relation to survival, but there was no significant difference across the groups in terms of overall survival and disease-free survival. This was not in compliance with the available data [11-14].

The method of surgery, either laparoscopy or open, did not have an impact on the survival outcome, in keeping with the published data [8]. In our study, the nodal yield and margin status were similar between the laparoscopic and open groups. The leak rate was also comparable, even though the number of leaks is of low incidence, making it difficult to draw conclusive results from it. This deems that minimally invasive surgery is an equally good option for surgery in rectal cancer patients.

The average nodal yield in the population is 7.13. This may be an effect of postfixation status, as a study from the same institution had given a mean nodal yield of 20 when the nodal tissue was dissected from a fresh ex vivo specimen. So, postfixation changes, as well as post-neoadjuvant therapy nodal fibrosis, may have led to the lower nodal yield. This may, in turn, have an impact on the survival, which needs further studies.

The pathological complete response in our study population was 11.1%, which was lower when compared to published data (13%-23%) [7,15-18]. The evaluation of the cause for the lower PCR rate is warranted. PCR was found to be significantly associated with longer OS, DFS, LRFI, and DMFI, but on multivariate Cox proportional hazard analysis, this significance was not seen. However, it is important to see that none of the patients with PCR had local recurrence.

Five percent CRM positivity was seen in the population, and this was a significant factor with an impact on OS, DFS, and DMFI. However, on multivariate Cox regression analysis, its significance was not seen. The distal margin was positive in 1% of the population; however, it did not translate into a detriment for overall survival or disease-free survival. Neither was there a difference in locoregional recurrence with respect to the length of the distal margin. This is in alignment with the consensus that post-NACTRT limited distal margin would be sufficient.

Positive lymph node ratio was determined to be an important prognostic factor, as it accounts for the total nodal yield to assess the burden of residual disease. With a cutoff of 0.28 based on data from various studies, it was noted to be significantly correlated with survival, in the form of OS, DFS, LRFI, and DMFI, which is in agreement with data from multiple previous studies [19-24].

Of the population, 97.44% had received adjuvant chemotherapy. The institutional protocol is to give adjuvant chemotherapy for all patients with rectal cancer post-NACTRT, except for patients who had achieved a pathological complete response. When comparing patients who had received chemotherapy to those who did not receive chemotherapy, there was no difference in outcomes, indicating that in selected cases, adjuvant chemotherapy may be avoided [25-27].

The clinically significant leak rate in the population studied was 2.2%. The impact of the leak on the oncological outcome was assessed, but no significant relation was determined. This analysis did not include occult leaks that may have occurred and may have been picked up by radiological imaging if the protocol for routine imaging was done. Hence, these data may be the tip of the iceberg and may not reliably predict the actual impact of leaks.

Of the population, 27.7% experienced disease recurrence during the study period. The majority of these were distant metastases. The pattern of recurrence in rectal cancer has traditionally been predominantly pelvic recurrence, but in our study, the data were contradictory to this [28]. This may be a reflection of better local control by radiation and the quality of TME combined. In this scenario, there is more reason to consider better systemic control as well in the form of chemotherapy or targeted therapy and also the implementation of the total neoadjuvant therapy, which is the only modality to have shown considerable overall survival. In the study population, two patients had undergone metastatectomy for metachronous metastasis, with survival similar to the disease-free population. However, one patient had undergone the re-excision of pelvic recurrence but had an early relapse. Hence, the aggressive radical management of distant diseases, whenever they arise, can contribute to better outcomes. With respect to local control, complete clearance is the only way to ensure freedom from local failure.

Limitations

This study is ambispective with the inherent limitations and bias in the retrospective component. Further, the data are from a single center; hence, the data may not be generalizable. Further, the molecular nature of the disease could not be studied during the study period, which may have an impact on survival. Selection bias also may have impacted the outcomes due to the retrospective nature of the study. Further, the study may have confounding factors that have impacted the survival, which could not be delineated.

It raises the possible ground for further research into the way we approach rectal cancer, with a possible question into the biological aspects of rectal cancer and regarding our management approaches and multimodality treatment. It validates the NAR and confirms its external validity in the Indian population and hence provides a useful tool to be used in further prospective trials and studies to estimate the beneficence of these approaches.

## Conclusions

The NAR score is a robust surrogate for the prediction of long-term outcomes and can be used to tailor an individual patient's management and also as a tool to validate and evaluate the effectiveness of various therapeutic options in a trial.

This opens a new avenue for further research into the nature of rectal cancer in the Indian population and to prompt evaluation into the optimal management in our population. This may include the intensification of systemic therapy, as it is the most common site of failure, and may include total neoadjuvant therapy. We have validated the short-term outcome and hope that this will lead to further robust studies.
